# COVID-19 and the Gendered Use of Emojis on Twitter: Infodemiology Study

**DOI:** 10.2196/21646

**Published:** 2020-11-05

**Authors:** Ahmed Al-Rawi, Maliha Siddiqi, Rosemary Morgan, Nimisha Vandan, Julia Smith, Clare Wenham

**Affiliations:** 1 Simon Fraser University Burnaby, BC Canada; 2 John Hopkins University Baltimore, MD United States; 3 Hong Kong University Hong Kong China (Hong Kong); 4 London School of Economics London United Kingdom

**Keywords:** emojis, social media, Twitter, gender, COVID-19, sentiment, meaning

## Abstract

**Background:**

The online discussion around the COVID-19 pandemic is multifaceted, and it is important to examine the different ways by which online users express themselves. Since emojis are used as effective vehicles to convey ideas and sentiments, they can offer important insight into the public’s gendered discourses about the pandemic.

**Objective:**

This study aims at exploring how people of different genders (eg, men, women, and sex and gender minorities) are discussed in relation to COVID-19 through the study of Twitter emojis.

**Methods:**

We collected over 50 million tweets referencing the hashtags #Covid-19 and #Covid19 for a period of more than 2 months in early 2020. Using a mixed method, we extracted three data sets containing tweets that reference men, women, and sexual and gender minorities, and we then analyzed emoji use along each gender category. We identified five major themes in our analysis including morbidity fears, health concerns, employment and financial issues, praise for frontline workers, and unique gendered emoji use. The top 600 emojis were manually classified based on their sentiment, indicating how positive, negative, or neutral each emoji is and studying their use frequencies.

**Results:**

The findings indicate that the majority of emojis are overwhelmingly positive in nature along the different genders, but sexual and gender minorities, and to a lesser extent women, are discussed more negatively than men. There were also many differences alongside discourses of men, women, and gender minorities when certain topics were discussed, such as death, financial and employment matters, gratitude, and health care, and several unique gendered emojis were used to express specific issues like community support.

**Conclusions:**

Emoji research can shed light on the gendered impacts of COVID-19, offering researchers an important source of information on health crises as they happen in real time.

## Introduction

### Background

COVID-19 has changed the way we communicate and interact with others. In an effort to maintain physical distancing and stop the spread of the virus, a lot of communication has moved from face-to-face (F2F) to online platforms including Twitter, Facebook, and Instagram, with users sharing information, messages, opinions, and beliefs about COVID-19 through these platforms. One form of online communication is through the use of emojis, defined as “a visual representation of an emotion, idea or symbolism. It conveys concern, humor, anger or sarcasm” [1]. Billions of emojis are sent every day in different social media platforms [2], indicating their widespread use and popularity. For instance, the face with tears emoji (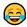) was named word of the year in 2015 and is considered the most used emoji across all genders [3].

This study focuses on how people of different genders (eg, men, women, and sex and gender minorities) are discussed in relation to COVID-19 through Twitter emojis. It asks the following research question: what are the gendered types of sentiments and meanings expressed with emojis with regard to the COVID-19 pandemic and how does emoji use differ when associated with different genders? We argue that, by exploring how the experiences of men, women, and sex and gender minorities in relation to COVID-19 are discussed using emojis, we can understand how emoji use represents or perpetuates (often inequitable) gender norms, roles, and relations in response to COVID-19, as well as how the pandemic may be impacting gender differently. Thus, analysis of emojis can provide a new methodological approach for rapid gender analysis in crisis settings, complementing more traditional forms of gender analysis through surveys or interviews.

The gendered dimensions of COVID-19 are becoming increasingly apparent. Although gendered dimensions of outbreaks have been demonstrated previously [4-6], given the widespread nature of COVID-19, these are becoming even more visible. First, women represent approximately 70% of health care workers worldwide and, thus, are disproportionately on the frontline of this battle against COVID-19 [7]. This formal care role is extended into homes, where informal care norms dictate that women provide care to sick family members—thereby exposing themselves to risk of infection and assuming the burden of work associated with this additional care [4]. This care burden also extends to the additional work created by having all family members at home during lockdown and the requirements for homeschooling as facilities remain shut. Women are increasingly seen to do this informal care work on top of their routine paid employment, with recent data demonstrating that women perform these domestic roles on top of paid employment considerably more than men, even when they are the main breadwinner in a household [8]. However, women are also disproportionately affected by job losses and furlough schemes, with data demonstrating that more women have been made redundant or furloughed than men [9]. This raises concerns for women’s economic empowerment and role in the labor force going forward. Women have also experienced increasing rates of domestic violence during lockdown, with calls to domestic violence hotlines surging in March and April 2020 when lockdown measures were enforced [10]. Finally, women’s differential health needs have been affected by COVID-19, with access to sexual reproductive health services and maternity care limited, reduced, or cancelled and deemed nonessential as resources are diverted to COVID-19 [11]. These illustrative examples demonstrate the vital need for gender analysis during outbreaks and in as real time as possible to outline the emerging gendered needs to policy makers throughout the crisis and postcrisis period.

Using emojis to relay ideas about health or disease is not new. For example, unique emojis have been used and introduced in relation to pandemics like malaria and Zika to raise awareness about their risks [12]. During the COVID-19 crisis when a shift from in-person conversations to a virtual communication paradigm occurred, emojis became even more vital to public discourse. For example, women in Ecuador are sending coded messages through emojis asking for assistance to escape domestic violence [13]. We also found many health-related emojis used in relation to all three gender groups, such as hospitalization (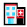), medical services (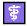), emergency sign (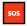), medicine (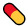), and syringe (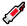). In addition, new types of emojis have emerged to represent key messages during the pandemic, including *wear a mask* (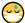) and *microbes* (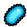), and a sequence of emojis provide specific social guidelines such as *do not sneeze into your hand* (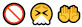) or *keep social distance* (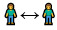). Another interesting aspect of emoji sequences is emphasis, as repeated use of one emoji like thank you (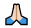) shows deeper appreciation than using the same emoji once. We know of no previous empirical research on the gender elements of health-related emoji use generally or on COVID-19 specifically.

### The Conceptualization of Emojis

Since the immense popularization of social media, coupled with the technological advancements in smartphone technology, emojis have witnessed an increasingly widespread use among different age groups and genders. Stark and Crawford [14] argued that emojis act as historical, social, and cultural objects, forming a type of a language that can help to underscore tone and communicate humor, allowing users to express their personality through their online interactions and relay a form of *digital feeling*. Emojis also allow users to express the characteristics that inform their individual identities including gender, race, age, and disability or demonstrate ideas or objects that are important to them. As such, exploring the use of emojis can provide important cultural and historical understanding into how people communicate, express themselves, or disseminate normative ideas and beliefs, especially during a public health crisis like the COVID-19 pandemic. In this study, we regard emojis as embodiments of affective expressions and “cultural objects” [14].

Generally speaking, the terms emojis and emoticons are often used interchangeably, although technically they connote different meanings. Although both are used as supplemental devices to nonvisual communication, emojis are pictorial representations, while emoticons are combinations of letters and punctuation marks available on smartphone and computer keyboards. For the purpose of this study, we treated both terms as interchangeable while concentrating our research interest mostly on emoji use. Emoticons resemble facial nonverbal behavior and may serve at least some of the same functions as nonverbal behavior in F2F communication [1].

Writing down emotional messages changes the intensity of the emotion because there is time to read over the text and reflect on one’s emotional state [15]. Emojis may enhance the exchange of emotional information by providing additional social cues beyond those found in a text message [16] used to augment the meaning of a message as a whole [17]. Emojis are also seen less as messaging tropes and more as expressive devices. Despite these strong positives for the use of emojis, there remains a perception in some quarters that their use is a lesser form of language and is devaluing and devolving language. However, these concerns are not only unfounded, but they ignore human needs for nonverbal communicative practices [18].

Emojis convey various forms of sentiments and messages that vary across cultures that need further scholarly attention. For example, in a multiphasic big data analysis based upon more than a million tweets and using Geert Hofstede’s national cultural scores, researchers established an unlikely relationship between cultures and vertical and horizontal emoticon use. In this regard, individualistic countries show a suppressed use of vertical emotions (emoticons emphasizing eye shape), whereas collectivistic cultures favor less horizontal emoticons (emoticons emphasizing mouth shape) [19]. Another more recent study reiterates the nonuniversality of emoticons through the analysis of selected populations of Tanzania, Cameroon, and Japan. Although Japanese people were sensitive to the different emotions embedded in emoticons, Cameroonian and Tanzanian people hardly read emotion from emoticons [20]. Further, Cheng [21] conducted an experiment that involved a study of sadness conveyed through both emoticons and emojis by Spanish and Chinese participants. The study confirmed that Spanish users prefer plain text messaging more than their Chinese counterparts, the latter registered a higher use of sad emojis and emoticons [21]. In brief, there are cultural differences in the way we use emojis and their types. Though it has not been empirically studied in previous research, many emojis are polysemous, as they can have more than one meaning, depending on cultural contexts and individual users.

### The Gendered use of Emojis

Although a plethora of research exists on emoji use itself, there is scarce scholarly knowledge that explores how gender norms, roles, and relations are represented within and perpetuated by emojis; instead, the bulk of previous research seems to be mostly focused on cross-cultural use of emojis, as previously indicated. During the beginning of emoji production, women were portrayed in stereotypical representations in emoji libraries; most activity-based emojis representing women were either brides or dancers, or exuded seductress characteristics [22]. Although the variations of smiley face emoji represent neither men or women, emojis were not always so gender neutral, though they were considered so when first released. For example, *neutral* images such as of a doctor or police officer were used to represent particular professions. The original emojis for such professions, however, represented the male body and clearly depicted men in these professions, reinforcing gendered norms and biases related to who engages in these types of professions. As Caroline Criado Perez [23] reports in her book, *Invisible Women*, what was striking was that it was not the original code that delineated these emojis as male but the platforms that interpreted gender-neutral terms as male.

In general, women were restricted to cliché portrayals while exempted from role depictions such as surgeons, lawyers, and teachers. This bias remained until 2016 when the original code was redesigned to gender all emojis. Today, male and female options exist for all professions and athletes, which is important because they act as a mechanism to perpetuate or reinforce inequitable gender norms, roles, and relations, which have had and continue to have a negative impact on peoples’ lives and health [24].

Most studies have shown varying results that women use emojis significantly more than men [25,26], and some scholars observe there are certain overlaps in the use patterns. Wolf [27], for instance, conducted research on a total of 251 posts on the USENET platform (a primitive form of data platforms used to read and distribute news posts) to examine behavioral patterns among women and men when it comes to emoji use. After determining the variety of the emoticons used, three main categories were established: smileys (positive), frowns (negative), and winks (sarcastic, funny, or flirting). In addition, the frequency of emoticon use was tabulated along gender use [27]. The results reiterated the idea of an emotional woman and an inexpressive man, and presented an interesting discussion about the blurring lines between the definitions of gender in emoji use, particularly the commonalities in employing humor by women and sarcasm by men [27]. In addition, Kalsoom and Kalsoom [22] used a semiotics approach and a feminist paradigm to map the meaning making potential of semiotic resources and critically examined stereotypical and professional women emojis. For example, the bunny girl emoji (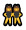) often refers to the objectification of women through a *showgirl* representation with costumes and dancing, while professional emojis highlight different professional roles such as scientist and doctor along different genders [22]. Danesi [28] uses a similar semiotic yet nontechnical approach to understand meaning making through signs and symbols of emojis, which he terms as a rather generic tool. On the other hand, an extensively thorough study of blogs used by people between the ages of 13-19 years revealed that men and boys have a tendency toward using emoticons coupled with active and resolute language, while women and girls used them to express strong social interactions [29].

Further, Kavanagh [30] collected posts from American and Japanese blogs and found that women’s emoticons were dominant in both high context culture (such as that of the Japanese), where communication is more indirect and symbolic, and low context cultures (such as America), where communication is more direct and succinct [30]. Further, Tossell et al [31] investigated how emoticons were used and, in particular, how gender differences exist in the frequency and variety of emoticons. For their analysis, data from 21 smartphones was taken over a 6-month period. In terms of quantity, the authors observed that women were more likely to use emoticons than men, while the latter preferred using a distinct range of emoticons to express themselves. Drawing on Tosell et al’s [31] findings, Shahbaz et al [32] conducted a study on the users of Kika Keyboard, a major Google play application with a diverse library of 1281 emojis. The authors found that “there are stark differences in the emoji usage preferences in men and women with women using more than one emoji in a single message and men using them in consecutive turns.” Another research conducted in 2018 at the Peking University in China commented extensively on the ubiquitous nature of emoticon use and its ability to surpass language barriers and travel worldwide. The study distinguishes between expressions among women and men, and suggests designing new gender-based machine learning modules [33].

Finally, diversifying the gender demographic in terms of age, Nishimura [34] conducted a qualitative study with 50 Japanese bloggers older than 60 years and men and women in their twenties and thirties to understand their emoticon use. The outcomes showed that younger women are more active in using emotions compared to other genders and age groups, followed by older women and older men. Young men in the age group of 20-40 years showed the lowest use of emoticons.

What is obviously lacking in previous research on gendered emojis is a focus on public health issues; hence, our study fills a gap in literature. In addition, research on sexual and gender minorities’ emoji use is still missing. This reveals a persistent pattern that excludes minority groups from scholarly representations of who is considered equal members within society. Buff [35] in her proposal to the Unicode consortium, which works to create universal international software standards, not only pushed for a much needed reform toward the introduction of gender-neutral signs but also the inclusion of the *third gender* in emojis, stressing the risks of solely highlighting gender binary models that can perpetuate harmful nonbinary gender stereotypes and outdated world views.

Our study attempts to explore how people of different genders are mentioned and discussed in connection to COVID-19 through the study of social media emojis.

## Methods

Through Twitter, we collected 50,811,299 tweets referencing #Covid-19 and #Covid19 that were posted by 11,706,754 unique users. The tweets were collected for a period of over 2 months from February 12 until April 18, 2020. We believed that the large number of tweets was enough to conduct our study on gendered emoji use because the data set contained a wide range of emojis, but it was not empirically possible to ascertain the sexual and gender backgrounds of social media posters, which remains a limitation in our study. This data set was collected using the TCAT platform that uses Twitter public application programming interface (API), allowing about 1% of public tweets to be fetched. Due to API limitations, the platform often hits the rate limit allowed, so a brief delay sometimes happens in collecting tweets. In other words, we have not violated the terms and conditions of the Twitter platform in our data collection. We then used several customized Python programming packages to first extract three gender-specific tweets (men, women, and nonbinary) and then fetch emojis from each data set (Table 1) [36].

**Table 1:** The top 20 most frequent emojis along gender groups.

No	Women	Frequency, n	Men	Frequency, n	Nonbinary	Frequency, n	All data	Frequency, n
1	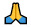	16,496	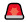	23,538	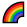	837	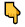	985,584
2	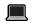	14,179	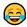	22,239	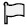	707	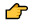	797,255
3	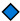	9732	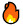	20,709	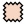	546	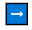	720,706
4	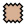	9347	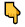	5894	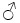	451	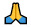	619,156
5	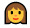	9187	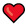	4338	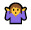	344	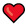	595,090
6	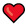	8783	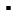	3933	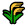	302	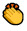	570,762
7	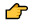	6281	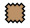	3078	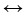	302	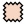	568,700
8	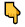	5718	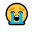	2740	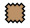	280	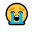	473,298
9	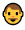	5574	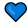	2723	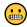	255	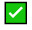	468,919
10	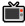	5461	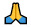	2602	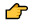	226	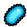	456,706
11	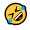	5376	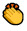	2460	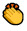	221	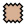	452,459
12	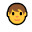	5054	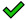	2163	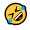	217	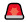	449,328
13	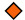	5035	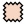	2018	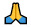	212	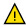	404,043
14	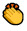	4655	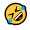	1900	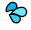	197	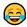	399,166
15	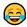	4502	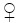	1775	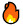	188	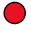	397,510
16	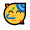	4303	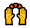	1416	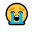	184	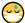	367,933
17	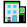	4251	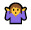	1408	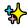	180	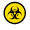	342,812
18	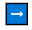	4166	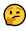	1219	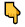	161	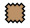	337,518
19	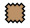	3726	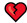	1137	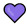	156	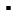	296,252
20	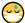	3513	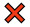	1091	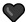	147	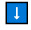	293,830

Though the search terms were not exhaustive and only relied on the English language, the search terms used to extract tweets on women were “woman*,” “women,” “femin*,” “girl*,” “lady,” “ladies,” and “female*,” and the total number of English language tweets referencing the aforementioned search terms was 541,698 tweets, constituting 1% of the total data set of 50,811,299 tweets. These filtered tweets were sent by 367,037 unique users. As for men, the search terms used were “boy*,” “man*,” “men,” “gentlem*,” and “mascul*,” and the total number of English language tweets referencing the aforementioned search terms was 297,155 tweets sent by 231,899 unique users. To gather data related to nonbinary genders and those often marginalized within binary gender constructs, we used the search terms “nonbinary,” “non-binary,” “trans,” “transgender,” “two spirited,” “two-spirited,” “LGBT*,” “gay*,” “homosexual*,” “lesbian,” “bisexual*,” and “queer,” which were classified under the heading “sexual and gender minorities” in the analysis that follows, which we recognized combines a vast range of gender and sexual identities. The total number of English language tweets referencing the aforementioned search terms was 26,048 posted by 20,744 unique users. These search terms were agreed upon after consultation with the research team members, and we recognize that they are not exhaustive, as gender identities and corresponding terms are constantly changing across various cultures.

Since the total number of extracted emojis were large in number (n=33,705,203) with 1297 different types, we focused on the top 600 emojis along the three data sets (the top 200 emojis representing each gender), using a manual approach in coding the emojis. We used a similar approach to Shahbaz et al [32] and Chen et al’s [33] studies, which focused on gender-based analysis of emoji use patterns including frequency, choice, and consecutive and discrete patterns. We believe that some of these patterns, especially the frequency of emojis, shed important light on the salience of certain public sentiments expressed on Twitter because the frequency indicates the salience given to some emojis or sentiments. For example, the significance of using one emoji thousands of times should be different from another emoji used only a couple of times.

The other strength of this approach to quantifying emojis is that it illustrates significant differences in the number of emojis used to represent the different identified genders (see Table 1), so it is more accurate to take into account when calculating the percentages of each gender. Though there are several automated methods to code emojis into their assigned sentiments [37], our study uses a combined novel approach of semiotic analysis to understand the connotative and denotative emoji expressions [22,28] and manual sentiment analysis [38].

To classify emojis into their assigned sentiments, we used the database of emoji sentiment rankings, which is based on Novak et al’s [39] emoji study. Their study investigated 1.6 million multilingual tweets (in 13 European languages), and human annotators manually classified and coded 751 different emojis found in these tweets by classifying them based on their sentiment ranking, indicating how positive, negative, or neutral each emoji was. In our study, we used negative, positive, and neutral values in measuring the sentiments of emojis instead of employing a Likert scale approach. This is because of the difficulty of obtaining intercoder reliability with a scalar approach, especially because the emojis we examined were uniquely related to the COVID-19 pandemic. For example, many emojis in our data sets were not listed in the aforementioned database. As a result, we used two other sources.

The first one was Unicode.org that lists more than 3300 emojis [40], and the second one is called emojipedia [41]. The latter is a consolidated database of most available emojis on modern smart devices, and it lists the varied meanings and interpretations of each emoji. These emoji repositories have been used in previous studies dealing with a variety of topics [42,43]. To make sure that our approach was valid and before we conducted the full scale study, two coders independently examined a representative 10% sample of the data sets (n=60), and the agreement between them was α≥.815 using Krippendorff alpha [44]. Finally, another novel approach that we followed in this study was examining some of the relevant emoji sequences because a combination of some emojis can denote a clearer message in the context of the pandemic, such as (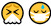), which means “if you are sick, wear a mask,” or (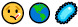), which denotes that “coronavirus has made many people sick around the world.” Other sequences include (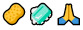), which refers to “please wash your hands before eating,” or (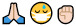), indicating “thank you or please wear a mask as an expression of solidarity.” To identify these emoji sequences, we used a customized Python script and searched in the three data sets.[36]

## Results

### Data

**Figure 1:**
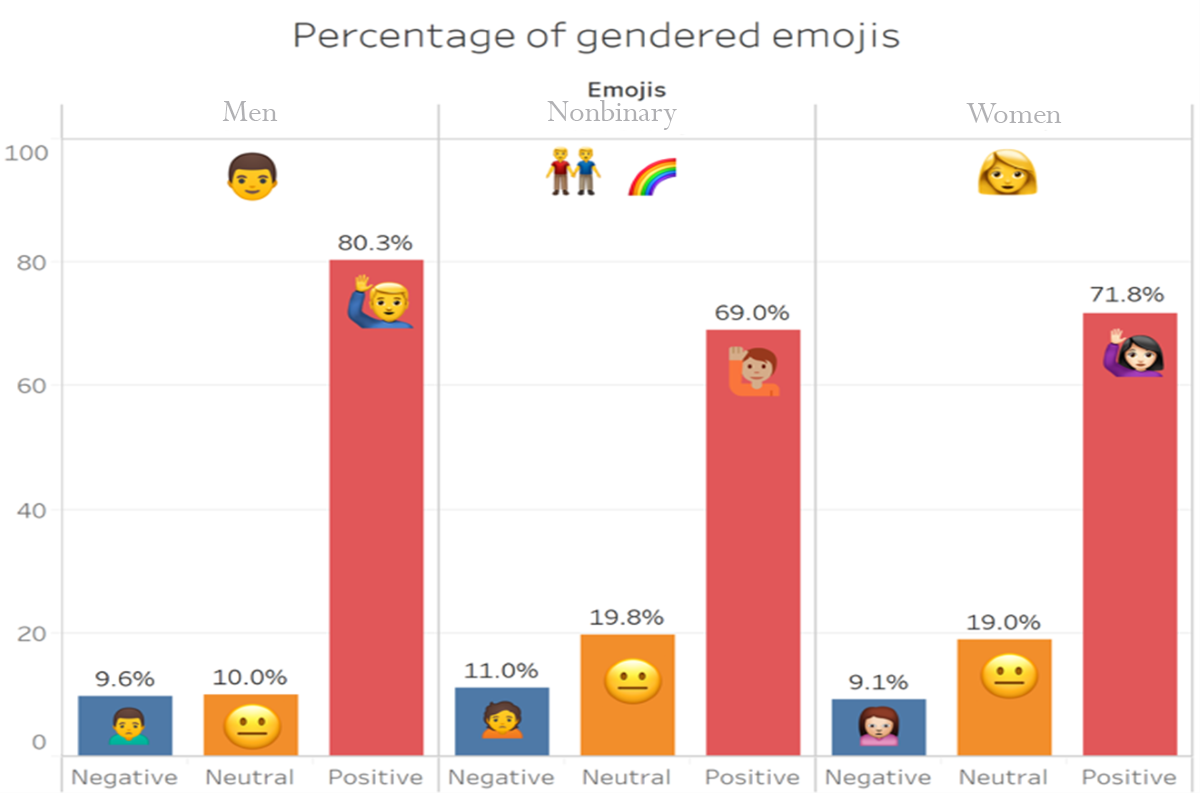
The percentage of emoji sentiments along the three main genders. This figure was generated using Tableau and Photoshop.

This study is focused on examining the gendered public discourses using emojis. To answer the study’s main research question, we found that gendered sentiments around COVID-19 were overwhelmingly positive alongside all genders, such as the predominant use of the *thank you* (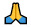) emoji (see Figure 1). Emojis used alongside discourses around men, however, showed a significant higher tendency toward using positive sentiments (112,516/140,056, 80.3%) as compared to the other two gendered groups: women (174,741/243,302, 71.8%) and sexual and gender minorities (8186/11,849, 69%). Yet emojis used alongside discourses around men and women showed similar trends when it came to the use of negatively ranked emojis (men: 13,476/140,056, 9.6%; women: 22,167/243,302, 9.1%) such as the *heartbreak* (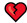) emoji compared to emojis used alongside discourses around sexual and gender minorities that showed the highest percentage of negativity (1315/11,849, 11%). As for neutral emojis, which neither indicate clear positive or negative sentiments like the neutral face (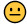) emoji, the analysis showed that discourses around sexual and gender minorities received the highest percentage (2348/11,849, 19.8%), followed by discourses on women (46,394/243,302, 19%), while the lowest use was found around discourses on men (14,064/140,056, 10%). In the following section, we present the five major themes that we qualitatively identified following the semiotic approach, which include morbidity and health fears, economic and employment concerns, health responses, support for frontline workers, and unique gendered emojis.

In the twitter data set, the total sum of the emoji counts (total number of times that emojis appeared) were as follows: 162,423 for men; 291,838 emojis referencing women; and 13,985 emojis mentioning sexual and gender minorities. The major themes that emerged from studying emojis based on their frequencies and percentages are discussed in the next sections.

### Morbidity and Health Fears

Twitter users employ different emojis to express fear, risk, and concerns that are noticeable in relation to the novel coronavirus, such as the coffin (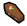) and skull (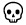) emojis, in reference to death, as well as the ambulance siren (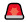) emoji, which often refers to the emergency siren vehicles carrying patients with COVID-19. In terms of gender differences, the coffin emoji was only found alongside discourses on men (n=129). This may be a reflection of how men and women are differentially perceiving the severity of COVID-19 due to sex differences in mortality among males and females, for male patients (in most contexts) are more likely to die due to COVID-19 compared to female patients [45]. In addition, the skull emoji was also mostly found in the discourses used around men (197/162,453, 0.12%) and, to a lesser extent, in relation to the discourses used around sexual and gender minorities (6/13,985, 0.04%). The siren emoji was used in the discourses around men (23,538/162,453, 14.7%) more than around women (2310/291,838, 0.8%) and sexual and gender minorities (56/13,985, 0.4%). Finally, the emoji sequence (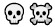) was only found in the men’s data set (n=37), and the emergency emoji sequence (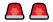) was the 12th most frequent sequence in the men’s data set. Another unique sequence in discussions about men was the following (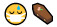), which is a warning that not wearing a face mask can lead to death.

### Economic and Employment Concerns

Another type of concern expressed online was related to financial matters. As the world experienced an economic crisis with uncounted layoffs and high unemployment rates during the pandemic, several emojis were used like money (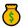) and bank (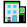) to indicate economic instability, lack of employment opportunities, limited access to money, or financial concerns. Here, we found that the money emoji was not used in the discourses around men, and it was present in the discussions related to sexual and gender minorities (19/13,985, 0.13%) and in those related to women (1868/291,838, 0.6%), yet the bank emoji was only found in the discourses around women (n=4251, 1.5%).

As a significant transition was made from in-person to online operations that entailed a work-from-home dynamic, we found that the emoji that portrayed this phenomenon was the PC (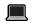), which was again mostly used in relation to discourses around women (n=14,179, 15%), and much less in relation to discourses around sexual and gender minorities (n=18, 0.16%) and men (n=88). Another relevant emoji that was only found in the discourses on women was the *working from home* emoji (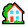; 496/291,838, 0.17%). The large difference in the use of these two latter emojis reflects the public discourses on the opportunities and challenges that women are facing during the pandemic. In Canada, for instance, women are more likely than men to hold jobs that can be performed from home during the pandemic [46]. These gender disparities shed light on the prominence of this emoji in the discourses on women.

### Health Responses

We also examined how online communities responded toward COVID-19 in relation to health considerations. For example, emojis were used to express health concerns like vomiting (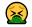), sneezing (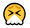), and fever (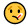), in addition to items that can be used to protect oneself from contracting COVID-19, such as the soap bar emoji (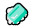) and face mask emoji (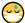). In relation to the gender differences, the soap emoji was not used in the discourses on men, but was found instead in the discourses around women (723/291,838, 0.2%) and, to a much lesser extent, discourses around sexual and gender minorities (8/13,985, 0.02%), while the face mask emoji was mostly used within discourses around women (3513/291,838, 1.2%), followed by men (660/162,453, 0.4%) and sexual and gender minorities (41/13,985, 0.2%).

In terms of the emoji sequence, we found that the women’s data set contained more health instructions and guidelines than the other two data sets. For example, the following two emojis (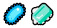; n=273) and (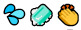; n=19) refer to washing with water and soap, which can help stop the virus, while (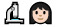) refers to “women’s testing” (n=59), (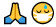) refers to “thank you or please wear a facemask” (n=42), and (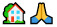) refers to “thank you or please stay at home” (n=22). Other sequences were longer, such as (
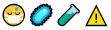
), which indicates “testing, wearing a mask, and taking cautious steps can stop the virus” (n=245). Although they were much less frequent, the following emoji sequences were found in the men’s data set: (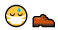), which signifies the need to wear a mask if you go outside, and (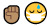) and (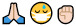), each showing solidarity for wearing the mask. Interestingly, the last two emoji sequences represent people from different racial backgrounds based on the skin tones used.

### Support for Frontline Workers

There was an interesting positive trend manifested in the emojis that showed support and appreciation for frontline health care workers, including the rainbow emoji (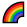) that is used to show solidarity with the lesbian, gay, bisexual, transgender, and queer community (see Table 1) but has also been recently employed to denote unity, togetherness, as well as good fortune during the COVID-19 pandemic. The use of this symbol to support frontline workers, however, has also created some concerns among sexual and gender minorities due to their need to use the flag to remind people of their causes and struggle to achieve equality [47]. Other positive emojis that express support for frontline workers included the applause emoji (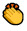), the folding hands as a thankful gesture emoji (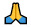), and the globe or global solidarity (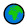) emoji. Here, we found some interesting gender differences in the thankful gesture, which was mostly used in discourses around women (16,496/291,838, 5.6%) compared to men (2602/162,423, 1.6%) and sexual and gender minorities (212/13,985, 1.5%). However, the applause emoji was used in similar percentages along the different genders (women: 4655/291,838, 1.6%; men: 2460/162,423, 1.5%; sexual and gender minorities: 221/13,985, 1.6%). Regarding the emoji frequencies, we found expressions of gratitude for hospital workers such as (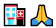; n=42) and (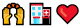; n=22) in the women’s data set.

### Unique Gendered Emojis

Finally, gender-specific emojis were often used in relation to women. For example, women were often represented through exercise such as doing yoga (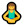), weight lifting (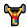), or running (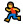). These emojis were not found in the top emojis examined in the study in the discourses around men or sexual and gender minorities, possibly indicating a higher concern and attention paid by women to maintain physical health during the pandemic. In addition, there were many public discussions related to pregnant women (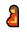; n=1441), breastfeeding (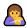; n=450), and a milk bottle (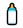; n=418), representing the unique challenges women face due to their reproductive role. Regarding emoji sequences, we found several combinations referring to the aforementioned activities, such as (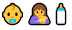; n=23), in the women’s data set.

## Discussion

### Principal Findings

Based on the previously mentioned findings on Twitter emojis, we found different discourses around men, women, and gender and sexual minorities. In general, emojis were positively associated with men while more negatively connected to sexual and gender minorities and women. In addition, there were unique emojis associated with women and the issue of health care workers as well as hygiene and with men and the issue of mortality.

Since the Twitter data was collected from different countries around the world, it is not possible to ascribe the aforementioned results to one country or culture, but we did find some familiar findings. For instance, there was a clear tendency to discuss men through positive sentiments, unlike the case of sexual and gender minorities, and to a lesser extent women, which might be related to the male-dominated Twitter platform that is estimated to be used by 62% men versus 38% women [48], as well as patriarchal cultural norms that span geographies. What is particularly notable here is the tendency to use negative emojis when discussing sexual and gender minorities, as this negativity appears to reflect ongoing stigma and discrimination toward these groups throughout recent history [49] and the way, for example, gay men were negatively affected by state policies in North America during the AIDS epidemic in the 1980s [50].

In addition, emojis offer insight into the COVID-19 impact on different genders. For example, we found a higher number of coffin and skull emojis when men were discussed, and generally, men have a higher mortality rate than women due to COVID-19. In addition, the financial burdens and concerns experienced by women, as well as the experiences of working from home, were reflected in the higher use of these emojis in relation to women. Interestingly, there is a prevalence in using the hand washing emojis in discussions about women, and there is evidence that responsibility for personal and family health and hygiene is most often born by women and that men are less likely to wash their hands or wear a face mask [51]. The more dominant use of emojis such as praying hands in relation to women may show appreciation for these health-related roles. As a matter of fact, the majority of frontline workers worldwide are women [52].

These findings indicate that the analysis of emoji use offers an innovative methodological approach to analyzing the gendered impacts of health crises like COVID-19. This is particularly important as there are frequent calls for rapid gender analysis of health emergencies [53], and research through more established means such as surveys and interviews can be slow and completed after the fact. Emojis present a readily available, real-time data source that depicts discourses on gendered impacts of health crises as they are happening. However, this is the first study, which we know of, to employ emoji analysis in this way. There is a need to further develop this method and expand the analysis in terms of both depth and scope.

### Conclusion

This study examined the gendered use of emojis on Twitter in relation to COVID-19, and the findings showed many differences alongside discourses of men, women, and gender minorities when certain topics were discussed, such as death, financial and employment matters, gratitude, and health care. In addition, there were several unique gendered emojis that were used to express specific issues like community support. In general, emojis are positively associated with men while more negatively connected to sexual and gender minorities and, to a lesser extent, women.

Our study is limited to the empirical examination of emoji use on Twitter in the context of COVID-19. It would be interesting to compare the emojified gendered public discourses on other platforms like Instagram and Facebook. Though it was not prominent in our top posts, emojis denoting race and ethnicity need to be incorporated as another important variable to be examined, and it would be interesting to conduct comparative studies on this aspect of emoji use. Subsequent studies might provide greater disaggregation of data related to sex and gender minority groups. Finally, our research is similar to previous studies on sentiment analysis that have limitations in terms of the dictionaries or word lists used [54] and the lack of examining the nature of users interacting online [55].

## Abbreviations

API: application programming interface
F2F: face-to-face
